# tRNA-Derived Fragment tRF-5009A Regulates Autophagy and Degeneration of Cartilage in Osteoarthritis via Targeting mTOR

**DOI:** 10.1155/2022/5781660

**Published:** 2022-08-05

**Authors:** Zengfa Deng, Dianbo Long, Hailong Liu, Yiyang Xu, Ruobing Xin, Hongyi Liao, Zhiwen Li, Ruiyun Li, Guping Mao, Ziji Zhang, Yan Kang

**Affiliations:** ^1^Department of Joint Surgery, The First Affiliated Hospital of Sun Yat-sen University, Guangzhou, Guangdong 510080, China; ^2^Guangdong Provincial Key Laboratory of Orthopedics and Traumatology, The First Affiliated Hospital of Sun Yat-sen University, Guangzhou, Guangdong 510080, China; ^3^Department of Orthopedics, Shengli Clinical Medical College, Fujian Provincial Hospital, Fujian Medical University, Fuzhou, Fujian 350000, China; ^4^Department of Anesthesiology, Guangdong Provincial People's Hospital, Guangdong Academy of Medical Sciences, Guangzhou, Guangdong 510080, China

## Abstract

tRNA-derived fragments (tRFs) have been reported to have critical regulatory roles in osteoarthritis (OA). Recent studies have suggested that autophagy promotes the homeostasis of the extracellular matrix of chondrocytes in OA. However, the role of tRFs in posttranscriptional gene regulation during autophagy in OA is unknown. Therefore, we explored the role of tRF-5009A in the posttranscriptional gene regulation of autophagy and cartilage degeneration in OA. Using RNA sequencing, we identified tRF-5009A, the tRNA^ValCAC^-derived fragment, in OA tissues and explored its expression by quantitative reverse transcription PCR and fluorescence in situ hybridization. We further investigated the relationship between the expression of tRF-5009A and clinical factors in OA. Chondrocytes were transfected with a tRF-5009A inhibitor or mimic to determine their functions, including in relation to autophagy and the cartilage phenotype. A rescue experiment and dual-luciferase reporter assay were conducted to determine whether the 3′-untranslated region (UTR) of mTOR contains a tRF-5009A-binding site. tRF-5009A was downregulated in the cartilage of OA knees, especially in damaged areas. mTOR was highly expressed in damaged cartilage and negatively correlated with the expression of tRF-5009A; transfection with a tRF-5009A inhibitor promoted the expression of mTOR and suppressed autophagy, whereas transfection with a tRF-5009A mimic had the opposite effect. A dual-luciferase reporter assay showed that tRF-5009A silenced the expression of mTOR by binding to its 3′-UTR. Thus, tRF-5009A regulates autophagy and cartilage degeneration in OA by targeting mTOR. In summary, these findings provide an additional tool for the clinical diagnosis and novel targeted therapy of OA.

## 1. Introduction

Osteoarthritis (OA) is a degenerative disease characterized by subchondral bone remodeling, joint inflammation, osteophyte formation, and cartilage degeneration [1, 2]. OA is a major cause of disability, and its incidence, which is related to sex, age, obesity, joint trauma, and other factors, is on the rise, bringing huge economic burden to patients and society [3, 4]. OA is primarily characterized by cartilage destruction and also involves pathological changes in other structures of the joint, including the meniscus, synovium, ligaments, fat pads, and surrounding tissues [1]. The pathogenesis of OA involves an imbalance in the redox state leading to oxidative stress in chondrocytes, aging, and apoptosis, as well as decreased anabolism and increased catabolism of the extracellular matrix [5, 6]. However, there are currently no effective therapeutic treatments for OA. Therefore, it is important to elucidate the pathogenesis and identify effective targets for the development of effective treatment strategies.

**Figure 1:**
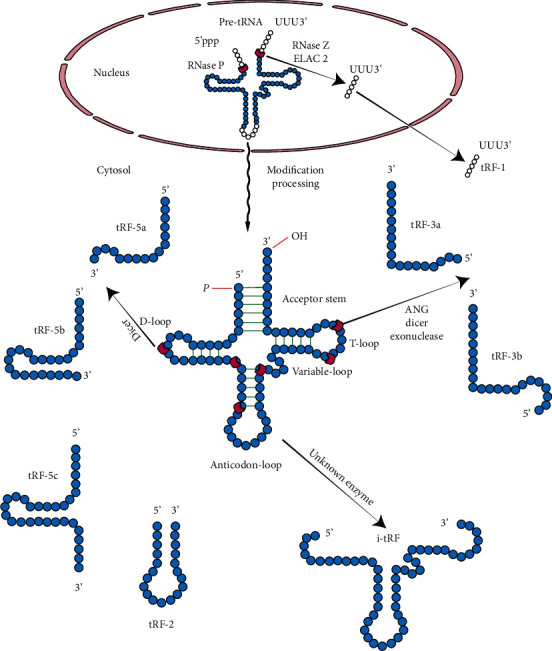
The structure of transfer RNAs (tRNAs) and principal categorization of tRNA-derived fragments (tRFs). tRFs are produced via tRNA anticodon loop cleavage under stress conditions. tRFs are divided into 5 subtypes, tRF-1, tRF-2, tRF-3, tRF-5, and i-tRF. tRF-1 results from pre-tRNAs processed by RNase Z, whereas tRF-2, tRF-3, tRF-5, and i-tRF are produced from mature tRNAs processed by Dicer, angiogenin (ANG), or other RNases at various sites.

In recent years, noncoding RNAs have become important regulatory factors in OA research. Transfer RNAs (tRNAs) are noncoding RNAs with distinct characteristics that function as key cellular translation factors, regulating a series of nontranslational processes [7]. tRNAs have strong secondary and tertiary structures and can be further processed into tRNA-derived fragments (tRFs) under stress conditions, including hypoxia, infection, and starvation. The origin, structure, and classification of tRNAs and tRFs are shown in Figure 1. In most organisms, tRFs with a length of approximately 14-30 nt are produced by the precise splicing of pre-tRNAs or mature tRNAs [8]. tRFs are divided into 5 subtypes: tRF-1, tRF-2, tRF-3, tRF-5, and i-tRF. tRF-1 results from the processing of pre-tRNAs by RNase Z. tRF-2, tRF-3, tRF-5, and i-tRF are produced from the processing of mature tRNAs by Dicer, angiogenin (ANG), or other RNases at various sites. tRFs might contain seed sequences that match the crosslinking central region of target mRNAs, resulting in mRNA silencing and posttranscriptional gene regulation [9, 10]. Currently, only a single study has been reported on the role of tRFs in OA, in which tRF-3003a was upregulated in IL-1*β*-stimulated chondrocytes, resulting in the downregulation of the expression of JAK3 [11].

The levels of inflammatory cytokines, oxidative stress, and endoplasmic reticulum stress are increased in OA, and several recent studies have strongly suggested that autophagy promotes the maintenance of the chondrocyte environment and extracellular matrix homeostasis [12–14]. The mammalian target of rapamycin (mTOR) is a key molecule in the mTOR signaling pathway and has been associated with many diseases, such as metabolic disorders and degenerative diseases [15]. The mTOR pathway acts on 2 different multiprotein complexes, mTOR complex 1 (mTORC1), which is associated with autophagy, cell growth, and proliferation, and mTOR complex 2 (mTORC2) [16]. The two mTOR complexes play very different roles in the regulation of metabolism and cellular proliferation [17]. mTORC1, composed of mTOR, Raptor, G*β*L, and DEPTOR, is inhibited by rapamycin. It is a major growth regulatory molecule that senses and binds to different nutritional and environmental factors, including growth factors, energy levels, cellular stress, and amino acids [18]. It binds these signals to promote cell growth by phosphorylating substrates to enhance anabolism (such as mRNA translation and lipid synthesis) or to limit catabolism (such as autophagy). Amino acid signaling directs the signal to mTORC1 in a manner independent of the PI3K/Akt axis and promotes the transport of mTORC1 to the lysosomal surface, where it interacts with Rheb and is activated [19]. The second complex, mTORC2, consists of mTOR, Rictor, G*β*L, Sin1, PRR5/ProTOR-1, and DEPTOR. mTORC2 promotes cell survival by activating Akt, regulates cytoskeletal dynamics by activating PKC*α*, and controls ion transport and growth by phosphorylation of SGK1 [20]. Several signaling pathways involving autophagy-activated mTOR have been reported in OA [21, 22]. In recent years, the mTOR autophagy pathway has been widely studied in OA. For example, a previous study revealed that moderate-intensity exercise promoted chondrocyte autophagy through the P2X7/AMPK/mTOR signal axis to alleviate pyroptosis, which provided novel insights into the positive and preventative effects of exercise on OA [21]. Besides, some researchers found that four-octyl itaconate improves osteoarthritis by enhancing autophagy in chondrocytes via PI3K/AKT/mTOR signaling pathway inhibition [23]. However, the underlying mechanisms of the function of tRF-5009A and mTOR in OA chondrocytes have not yet been elucidated. In this study, we aimed to determine whether tRFs regulate autophagy in OA chondrocytes.

## 2. Materials and Methods

### 2.1. Patient Recruitment, Sample Collection, and Cell Culture

This study was approved by the Clinical Research Ethics Committee of the First Affiliated Hospital of Sun Yat-sen University ([2021]334). Informed consent was obtained from all participants. Osteoarthritis articular tissues were acquired from patients (*n* = 6; mean age: 67 years; range: 60–72 years; male: 2, female: 4) with knee OA who underwent total knee arthroplasty, whereas normal articular tissues were obtained from patients (*n* = 6; mean age: 32 years; range: 21–45 years; male: 3, female: 3) who underwent lower limb amputation due to trauma without OA or rheumatoid arthritis. Collected articular tissues included the cartilage, meniscus, anterior cruciate ligament, synovial membrane, and infrapatellar fat pad. In addition, 30 cartilage specimens and clinical data of patients with knee OA who underwent total knee arthroplasty were collected and divided into the undamaged (ICRS = 0) and damaged (ICRS = 1–4) groups according to the International Cartilage Repair Society (ICRS) grading system [24]. Chondrocytes, infrapatellar fat pad cells [25], meniscus cells [26], anterior cruciate ligament cells [27], and synovial membrane cells [28] were isolated and cultured as previously described.

### 2.2. RNA Extraction and qRT-PCR

Total RNA was extracted using an RNeasy mini kit (Qiagen, Germany) according to the manufacturer's instructions. Extracted RNA was quantitatively evaluated using a NanoDrop spectrophotometer (NanoDrop Technologies, USA). The rtStar tRF and tiRNA pretreatment kit (Cat. #AS-FS-005; Arraystar) was used to preprocess the miRNA samples. cDNA was synthesized using the rtStar first-strand cDNA synthesis kit with 3′ and 5′ adaptors (Cat. #AS-FS-003; Arraystar). qRT-PCR was performed using an ABI ViiA™ 7 real-time PCR system (ViiA 7Dx; Applied Biosystems, USA). Primer sequences are listed in Supplementary Table S1. *GAPDH* was used as the reference gene for the evaluation of mRNA expression, while the U6 small nuclear RNA was used as the reference gene for the evaluation of expression of tRFs. The 2^−*ΔΔ*Cq^ method was used to calculate the levels of gene expression [29], and each experiment was repeated 6 times.

### 2.3. Target Gene Prediction and Bioinformatic Analysis

tRF sequencing data from a previous study are available on this website (https://www.ncbi.nlm.nih.gov/geo/query/acc.cgi?acc=GSE200433) [27]. miRanda and TargetScan were used to explore potential target genes of tRF-5009A using online analysis tools. Gene Ontology function analysis (http://www.geneontology.org/) and Kyoto Encyclopedia of Genes and Genomes (KEGG) pathway analyses were used to predict the pathways for tRF-5009A.

### 2.4. Transfection Using the tRF-5009A Inhibitor and Mimic and mTOR Knockdown (KD) shRNAs

Cells were seeded in 6-well plates and cultured to approximately 80% confluence and then transfected with either a *Homo sapiens* tRF-5009A inhibitor or mimic (RiboBio, Shanghai, China) or mTOR KD shRNAs (Tsingke Biotechnology Co., Beijing, China) using Lipofectamine 3000 (Invitrogen, USA) according to the manufacturer's instructions. Cells were collected for qRT-PCR after 24 h and for western blotting after 48 h. Nonspecific tRF (RiboBio) and KD NC shRNA (Tsingke Biotechnology Co.) were used as negative controls (NCs).

### 2.5. Histology, RNA Fluorescence In Situ Hybridization, and Immunofluorescence

After fixing in 4% paraformaldehyde (Sigma-Aldrich, St. Louis, MO, USA) and embedding in paraffin, 5 *μ*m sagittal sections of cartilage specimens were cut and stained with hematoxylin and eosin (H&E), safranin O solution, and toluidine blue (Sigma-Aldrich) for histological examination. After washing with PBS (Servicebio, Wuhan, China), chondrocytes grown on slides were fixed with 4% paraformaldehyde for 30 min. Cells were then cocultured with a fluorescence in situ hybridization probe targeting tRF-5009A. After washing with PBS, cells were stained with DAPI. Images were captured using a confocal microscope (LSM780; Carl Zeiss, Germany). Immunofluorescence was performed 48 h after transfection, as previously reported [30]. The primary antibody used was against mTOR (1 : 100; Cell Signaling Technology, Danvers, MA, United States), while the conjugated secondary antibody (Cell Signaling Technology, Danvers, MA, United States) was a goat anti-rabbit IgG. Images were obtained using a confocal laser microscope (LSM 780, Zeiss) at different magnifications.

### 2.6. Transmission Electron Microscopy

Transmission electron microscopy (TEM) was performed 48 h after transfection. Cartilage cells were fixed with 2.5% glutaraldehyde (pH 7.4; Sigma-Aldrich) for 2 h at 25°C. After centrifugation at low speed (1200 rpm, 3 min), a mung-bean-sized cell mass was observed at the bottom of the tube. Cells were treated and fixed overnight in 2.5% glutaraldehyde and after 2 h harvested in 1% osmium tetroxide (Structure Probe, Inc, USA) at 4°C. Following dehydration, infiltration, embedding, and sectioning, images were captured using a Tecnai G2 Spirit Twin transmission electron microscope (FEI Company, USA).

### 2.7. GFP-mRFP-LC3 Adenovirus Infection

Chondrocytes were inoculated on cell-climbing slides and transfected with GFP-mRFP-LC3 adenovirus (multiplicity of infection (MOI) = 50; Hanbio, Shanghai, China) before cell transfection. Cells were fixed in 4% paraformaldehyde for 30 min at 25°C, and LC3-puncta were detected using a NIKON Eclipse Ti confocal microscope (Tokyo, Japan).

### 2.8. Western Blotting

Western blotting was performed as previously described [31]. Primary antibodies against mTOR (1 : 1000; Cell Signaling Technology), ULK1 (1 : 500; Proteintech, Wuhan, China), p-ULK1 (1 : 2000; Proteintech), ATG13 (1 : 500; Servicebio, Wuhan, China), Beclin1 (1 : 1000; Proteintech), p62 (1 : 500; Servicebio), LC3 (1 : 1000; Servicebio), MMP13 (1 : 500; Servicebio), COL2A1 (1 : 1000; Abcam, Cambridge, UK), and GAPDH (1 : 3,000; Cell Signaling Technology) were used. Protein bands were detected using a chemiluminescence system (Bio-Rad Laboratories, Hercules, CA, United States) with an enhanced chemiluminescence (ECL) kit (Millipore, Darmstadt, Germany). Signal intensity was compared using the ImageJ software (NIH, Bethesda, MD, USA).

### 2.9. Flow Cytometry

Cell apoptosis was detected using the FITC annexin V apoptosis detection kit I (BD Biosciences, USA) according to the manufacturer's instructions and examined using flow cytometry (Beckman Coulter, USA). Results were analyzed using the FlowJo software.

### 2.10. Quantification of Reactive Oxygen Species

ROS detection kits (Beyotime) were used to determine and evaluate the production of ROS according to the manufacturer's instructions. Samples were examined using an inverted fluorescence microscope (Leica, Germany), and fluorescence intensity was detected using the ImageJ software.

### 2.11. Luciferase Reporter Assay

To detect the regulatory relationship between tRF-5009A and mTOR mRNA, a dual-luciferase reporter assay was performed using SW1353 cells (a human chondrosarcoma cell line). The 3′-untranslated region (UTR) of mTOR mRNA with the tRF-5009A-binding site or its mutant construct was inserted into a luciferase reporter plasmid (Promega, USA). The luciferase reporter was cotransfected with the mTOR 3′-UTR fusion vector and the tRF-5009A mimic or corresponding NC into SW1353 cells. Cells were collected 48 h later and subjected to a dual-luciferase reporter assay (Promega, USA) using a Synergy H1 microplate reader (BioTek Instruments, USA) for the detection of firefly and Renilla luciferase activities.

### 2.12. Statistical Analysis

Data were analyzed using GraphPad Prism 8.0 (GraphPad, La Jolla, CA, USA) or SPSS v26.0 (IBM Corp., USA). The Shapiro-Wilk normality test was used to explore data distribution. For values with normal distribution, unpaired *t*-tests for 2 groups and one-way analysis of variance (ANOVA) for multiple groups were used. The Mann–Whitney test for 2 groups and Kruskal-Wallis test for multiple groups were used for the analysis of differences in values with nonnormal distribution. The relationship between tRF-5009A and mTOR was analyzed using simple linear regression. The relationships between tRF-5009A or mTOR expression and baseline characteristics of patients with OA were evaluated using the chi-squared test and Spearman's correlation analysis. *R*^2^ < 0.16 means a low linear correlation; 0.16 ≤ *R*^2^ < 0.49 means a significant correlation; 0.49 ≤ *R*^2^ < 1 means a high linear correlation. *p* < 0.05 was considered significant.

## 3. Results

### 3.1. tRF-5009A Was Downregulated in Cartilage of Osteoarthritic Knee, Especially in the Damaged Area, and Associated with Clinicopathological Features

**Figure 2:**
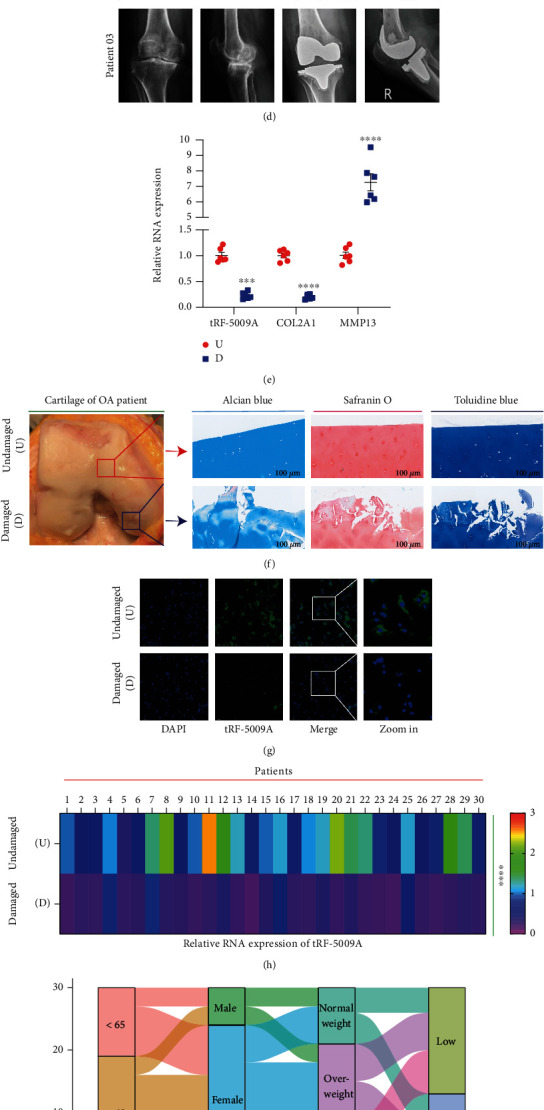
The expression of tRF-5009A was downregulated in the cartilage of knees with osteoarthritis (OA), especially in IL-1*β*-treated and damaged area cells. (a) The expression of tRF-5009A was downregulated most significantly in the cartilage compared with that in meniscus, anterior cruciate ligament, synovial membrane, and infrapatellar fat pad of OA knees. (b) The RNA expression of tRF-5009A, COL2A1, and MMP13 in OA and IL-1*β*-treated OA cells. (c) Structure and sequence of tRF-5009A, derived from tRNA^ValCAC^. (d) Plain radiograph (*n* = 3). A-P: anterior-posterior. (e) The RNA expression of tRF-5009A, COL2A1, and MMP13. (f) Alcian blue, safranin O, and toluidine blue staining. (g) RNA fluorescence in situ hybridization analysis of tRF-5009A in undamaged (U) and damaged (D) OA cartilage. (h) Heatmaps of relative RNA expression of tRF-5009A in undamaged (U) and damaged (D) areas (*n* = 30). (i, j) Relationship between age, sex, obesity grade, affected side, disease duration, obesity grade, Kellgren-Lawrence grade, and expression of tRF-5009A in the damaged area represented by the Sankey diagram. All data were expressed as the mean ± SEM. ^∗∗^*p* < 0.01,  ^∗∗∗^*p* < 0.001, and^∗∗∗∗^*p* < 0.0001.

In our previous study, tRF array analysis and qRT-PCR showed that tRF-5009A was downregulated in OA anterior cruciate ligament (ACL) cells [27]. To evaluate the potential role of tRF-5009A in OA, we first explored the expression of tRF-5009A in cartilage, meniscus, ACL, synovial membrane, and infrapatellar fat pad. Our qRT-PCR results showed that the expression of tRF-5009A was downregulated the most in the cartilage of OA knees compared with that in normal (Figure 2(a)). Therefore, we selected the cartilage for subsequent analysis of tRF-5009A. Next, we compared the relative RNA expression of tRF-5009A, COL2A1, and MMP13 in OA and in OA with IL-1*β*-treated cells (Figure 2(b)). The tRF-5009A sequence was derived from the 5′-end of tRNA^ValCAC^ (Figure 2(c)). To better study the potential role of tRF-5009A in OA cartilage, we collected cartilage samples from 30 patients with knee OA and divided them into undamaged (U) and damaged (D) areas based on preoperative radiography (Figure 2(d)). Imaging of surgical specimens, alcian blue, safranin O, and toluidine blue staining showed that the undamaged cartilage appeared smooth, whereas the damaged cartilage had defects and cracks (Figure 2(f)). Immunofluorescence showed that the expression of tRF-5009A was clearly reduced in the damaged compared with that in the undamaged cartilage (Figure 2(g)). We further detected the relative RNA expression of tRF-5009A, COL2A1, and MMP13 using qRT-PCR. We found that the expression of tRF-5009A was considerably downregulated in the damaged cartilage of patients with knee OA (Figures 2(f) and 2(h)).

To explore the clinical significance of tRF-5009A in OA, we used Sankey diagrams to present the relationships between the levels of tRF-5009A in the damaged area and patient characteristics, including age, sex, obesity grade, affected side, disease duration, and Kellgren-Lawrence grade (Figures 2(i) and 2(j)). We observed that among the 30 patients with OA, 17 had low expression of tRF-5009A, whereas 13 had high expression in the damaged area (Table 1). Chi-squared tests indicated that the low expression of tRF-5009A was significantly correlated to the Kellgren-Lawrence grade (*p* < 0.001) (Table 2). In addition, Spearman's correlation analysis showed that the low expression of tRF-5009A was associated with weight (*p* = 0.040), body mass index (BMI; *p* = 0.006), obesity grade (*p* = 0.008), and Kellgren-Lawrence grade (*p* < 0.001) (Table 3).

### 3.2. Prediction of the Target Gene of tRF-5009A

**Figure 3:**
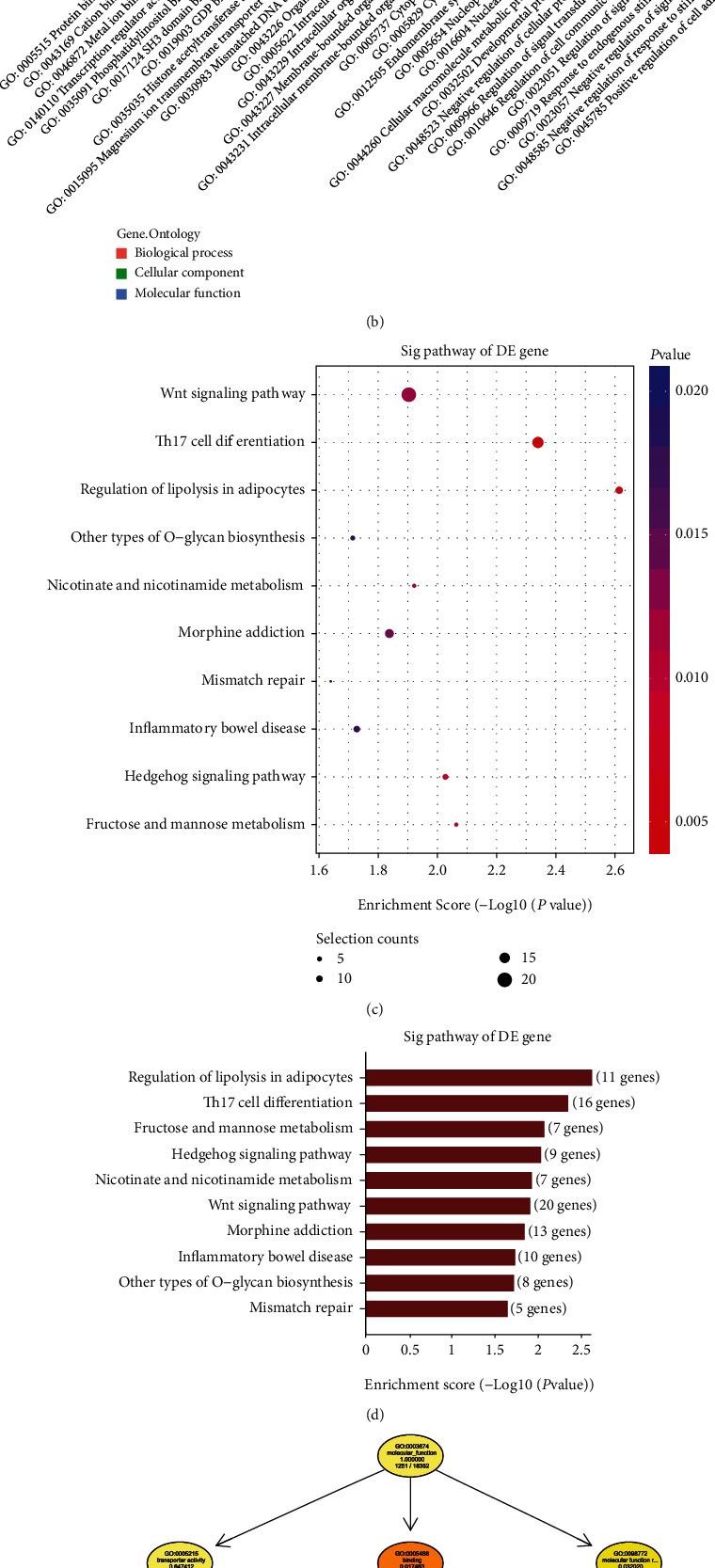
Bioinformatic analysis of tRF-5009A. (a) Barplot showing Gene Ontology (GO) enrichment for biological processes with top 10 scores. (b) Barplot showing significant GO terms as enrichment score values of significantly enriched pathways with top 10 scores for biological processes, cellular components, and molecular functions. (c) Dotplot showing gene ratios in the pathways with top 10 scores. The size of the dot indicates the number of genes with biological process terms, while the color represents the *p* value. (d) Barplot showing significantly enriched pathways with top 10 scores. (e) GO-directed acyclic graph explanation. When a gene is annotated to a specific node, it is also considered annotated to the parent nodes.

We first used TargetScan and miRanda to predict possible targets of tRF-5009A. We further used GO and KEGG pathway analyses to predict the functional enrichment and pathways of the assumed target genes. Our results revealed that *mTOR* was a potential target gene of tRF-5009A (Figures 3(a)–3(e)).

### 3.3. Expression of mTOR and Correlation between mTOR and tRF-5009A in Undamaged (U) and Damaged (D) Cartilage of OA Knee

**Figure 4:**
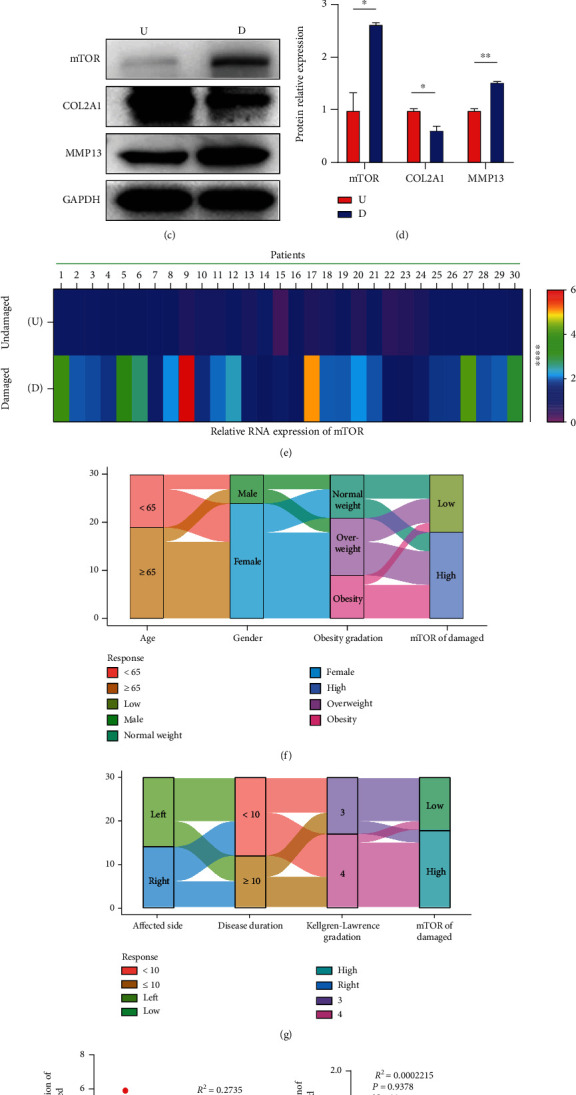
Expression of mTOR and correlation between tRF-5009A and mTOR in undamaged (U) and damaged (D) cartilage of OA knee. (a, b) Expression of mTOR detected by immunofluorescence and qRT-PCR in undamaged (U) and damaged (D) OA cartilage. (c) Western blotting of mTOR, COL2A1, and MMP13. (d) Quantification analysis of western blotting results in undamaged (U) and damaged (D) areas. (e) Quantification using heatmaps for the relative protein expression of mTOR in undamaged (U) and damaged (D) areas (*n* = 30). (f, g) Relationship between age, sex, obesity grade, affected side, disease duration, obesity grade, Kellgren-Lawrence grade, and expression of mTOR in the damaged area represented by the Sankey diagram. (h, i) Linear regression analysis of the expression of tRF-5009A and mTOR, respectively, in damaged (D) and undamaged (U) areas (*n* = 30). All data were presented as the mean ± SEM. ^∗^*p* < 0.05,  ^∗∗^*p* < 0.01, and^∗∗∗∗^*p* < 0.0001.

To evaluate the role of mTOR in OA, we examined the expression of mTOR in undamaged (U) and damaged (D) cartilages. We found that mTOR was highly expressed at both mRNA and protein levels in damaged compared with those in undamaged areas (Figures 4(a)–4(d)). To further investigate the clinical significance of the expression of mTOR and the correlation between mTOR and tRF-5009A in OA, we also tested the relative RNA expression of mTOR in the undamaged (U) and damaged (D) cartilage of 30 patients with knee OA (Figure 4(e)). We specifically detected that among the 30 patients with OA, 12 had low expression, whereas 18 had high expression of mTOR in the damaged cartilage (Table 1). The relationships between the expression of mTOR in the damaged area of the cartilage with patient characteristics, including age, sex, obesity grade, affected side, disease duration, and Kellgren-Lawrence grade are presented in the Sankey diagram in Figures 4(f) and 4(g). We further found that the expression of mTOR was negatively correlated with that of tRF-5009A in the damaged cartilage (Figure 4(h)), whereas no significant correlation was observed in the undamaged cartilage (Figure 4(i)). Chi-squared tests showed that the high levels of mTOR were associated with Kellgren-Lawrence gradation (*p* < 0.001) (Table 4). Finally, Spearman's correlation analysis revealed that the high levels of mTOR were significantly correlated with weight (*p* = 0.044), BMI (*p* = 0.010), obesity grade (*p* = 0.013), and Kellgren-Lawrence grade (*p* < 0.001) (Table 5).

### 3.4. tRF-5009A Inhibitor Suppressed Autophagy and Promoted Degeneration of OA Cartilage

**Figure 5:**
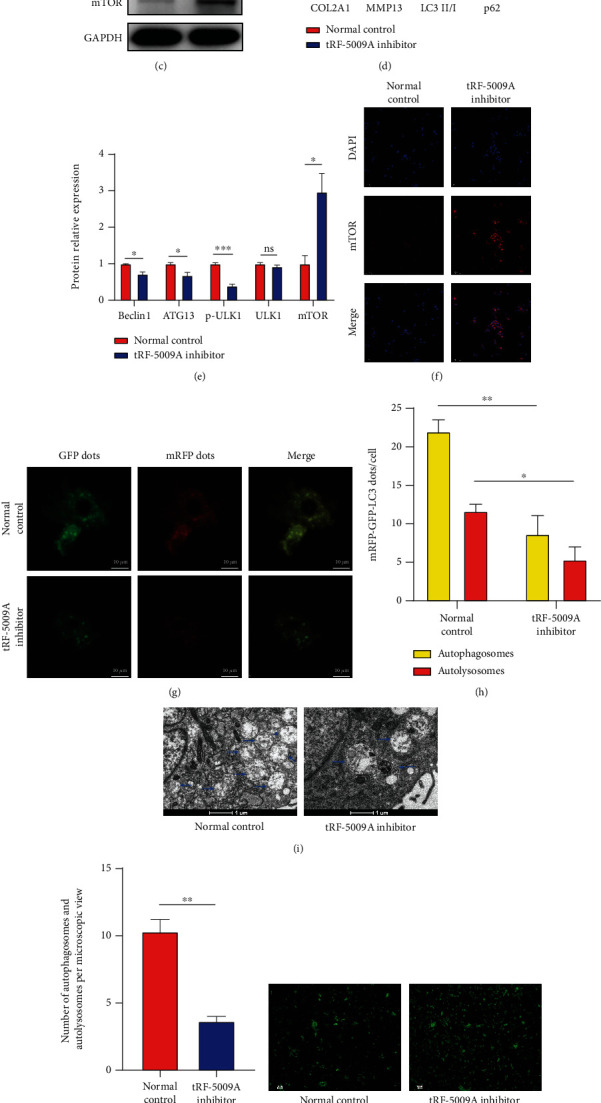
tRF-5009A inhibitor suppresses autophagy and promotes degeneration of OA cartilage. (a) Inhibitor efficiency of tRF-5009A by qRT-PCR. (b) qRT-PCR analysis of the expression of mTOR, COL2A1, and MMP13 after 24 h of OA cell transfection with a tRF-5009A inhibitor. (c) Western blotting results of COL2A1, MMP13, LC3, p62, Beclin1, ATG13, p-ULK1, ULK1, and mTOR. (d, e) Quantification analysis of western blotting results. (f) Expression of mTOR detected by immunofluorescence. (g) Double labelling with mRFP-GFP-LC3 adenovirus and (h) quantification of mRFP and merge dots per cell in chondrocytes. mRFP (red) represents autolysosomes (ALs); merge (yellow) represents autophagosomes (APs). (i) Transmission electron microscopy (TEM) analysis (blue arrows = APs and ALs) and (j) quantitative analysis. (k) Levels of reactive oxygen species (ROS) analyzed by DCFH-DA staining and (l) quantification analysis of mean fluorescence intensity (MFI). (m) Evaluation of cell apoptosis rate by flow cytometry. All data were expressed as the mean ± SEM. ns: not significant, ^∗^*p* < 0.05,  ^∗∗^*p* < 0.01,  ^∗∗∗^*p* < 0.001, and^∗∗∗∗^*p* < 0.0001.

To further investigate whether tRF-5009A regulates the expression of mTOR in OA cartilage, we first transfected cells with a tRF-5009A inhibitor and found that it decreased the expression of tRF-5009A (Figure 5(a)). We further detected that the expression of mTOR was significantly upregulated at both the mRNA and protein levels after transfection of cells with a tRF-5009A inhibitor (Figures 5(b)–5(f)). Moreover, we found that the levels of autophagy-related protein markers downstream of mTOR, including p-ULK1, ATG13, Beclin1, p62, and LC3 II/I, were also accordingly changed (Figures 5(c)–5(e)). We specifically noticed that COL2A1, a protective cartilaginous marker, was significantly downregulated at both the mRNA and protein levels, whereas MMP13, a destructive marker, was significantly upregulated (Figures 5(b)–5(d)). Transmission electron microscopy (TEM) following double labelling with mRFP-GFP-LC3 adenovirus showed that the levels of autolysosomes (ALs) and autophagosomes (APs) were downregulated after treatment with the tRF-5009A inhibitor (Figures 5(g)–5(j)). Moreover, DCFH-DA staining showed that the levels of ROS were increased, while flow cytometry analysis revealed that cell apoptosis was also increased (Figures 5(k)–5(m)). Together, these results suggested that the tRF-5009A inhibitor suppressed autophagy and promoted the degeneration of OA cartilage by regulating mTOR.

### 3.5. tRF-5009A Overexpression Promoted Autophagy and Suppressed Degeneration of OA Cartilage

**Figure 6:**
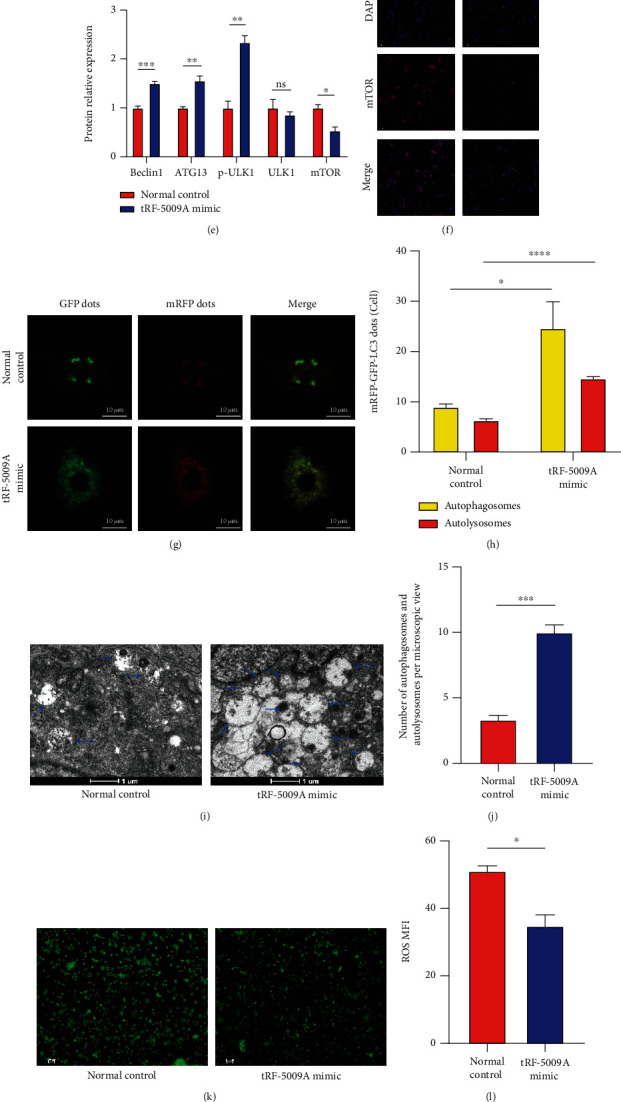
Overexpression of tRF-5009A promotes autophagy and suppresses degeneration of OA cartilage. (a) Overexpression efficiency of tRF-5009A by qRT-PCR. (b) qRT-PCR analysis of the expression of mTOR, COL2A1, and MMP13 after OA cell transfection with a tRF-5009A mimic. (c) Western blotting results of COL2A1, MMP13, LC3, p62, Beclin1, ATG13, p-ULK1, ULK1, and mTOR. (d, e) Quantification analysis of western blotting results. (f) Expression of mTOR detected by immunofluorescence. (g) Double labelling with mRFP-GFP-LC3 adenovirus and (h) quantification of mRFP and merge dots per cell in chondrocytes. mRFP (red) represents autolysosomes (ALs); merge (yellow) represents autophagosomes (APs). (i) Transmission electron microscopy (TEM) analysis (blue arrows = APs and ALs) and (j) quantitative analysis. (k) Levels of reactive oxygen species (ROS) analyzed by DCFH-DA staining and (l) quantification analysis of mean fluorescence intensity (MFI). (m) Flow cytometry analysis. All data were expressed as the mean ± SEM. ns: not significant, ^∗^*p* < 0.05,  ^∗∗^*p* < 0.01,  ^∗∗∗^*p* < 0.001, and^∗∗∗∗^*p* < 0.0001.

To further identify the role of tRF-5009A in the treatment of OA, we transfected OA cartilage cells with a tRF-5009A mimic and found that it significantly increased the expression of tRF-5009A (Figure 6(a)). We also detected that the expression of mTOR was downregulated, and the levels of its downstream autophagy-related protein markers were accordingly significantly altered (Figures 6(b)–6(f)). In addition, we found that the levels of the COL2A1 protective marker were upregulated, whereas those of the MMP13 destructive marker were significantly downregulated (Figures 6(b)–6(d)). TEM following double labelling with mRFP-GFP-LC3 adenovirus showed that the levels of ALs and APs were increased (Figures 6(g)–6(j)), whereas those of ROS and apoptosis decreased after overexpression of tRF-5009A (Figures 6(k)–6(m)). These results suggested that overexpression of tRF-5009A promoted autophagy and suppressed OA cartilage degeneration by regulating mTOR.

### 3.6. Effects of mTOR Knockdown on OA Cartilage

**Figure 7:**
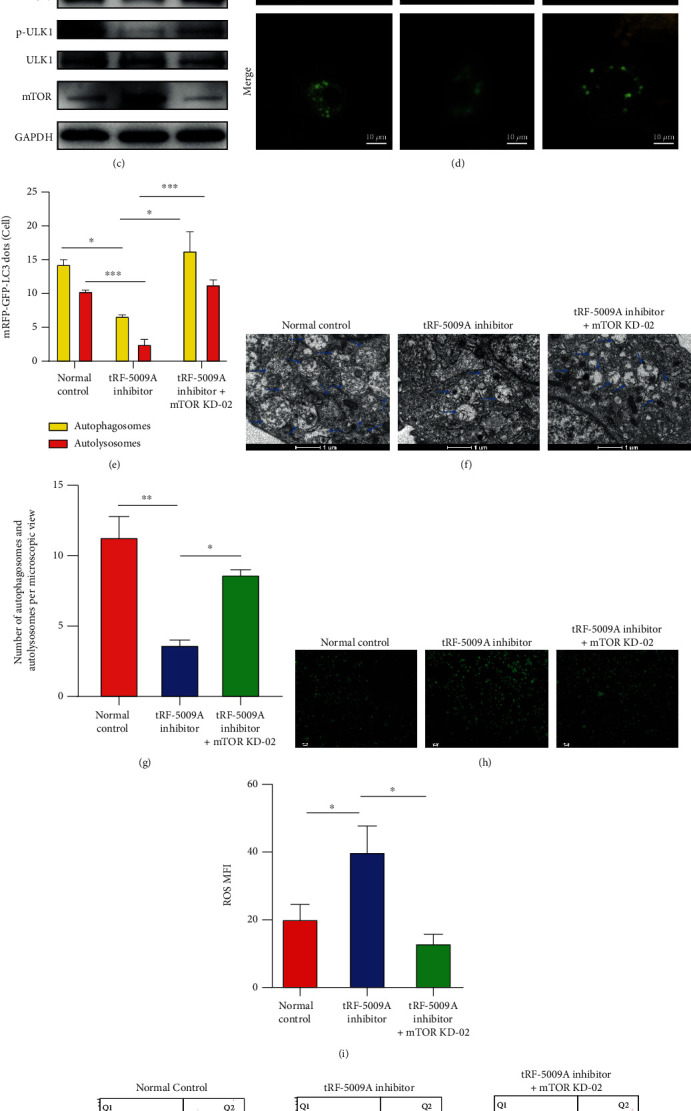
Effects of tRF-5009A binding to the 3′-UTR of mTOR on translation. (a) qRT-PCR analysis of the expression of mTOR, COL2A1, and MMP13 in OA cells transfected with mTOR knockdown (KD) shRNAs or negative control (NC). (b) qRT-PCR, (c) western blotting, (d) mRFP-GFP-LC3 adenoviral double labelling, (f) transmission electron microscopy (TEM), (h) levels of reactive oxygen species (ROS), and (j) flow cytometry evaluation of the effects of cotransfection of cells with a mTOR KD-02 and a tRF-5009A inhibitor. (e) Quantification analysis of mRFP (red) and merge (yellow) dots per cell. (g) TEM images and (i) mean fluorescence intensity (MFI) of ROS. (k) Schematic illustration showing the flowchart of the prediction of the tRF-5009A-binding site in *mTOR* using a mutant. (l) Relative luciferase activity. All data were presented as the mean ± SEM. ns: not significant, ^∗^*p* < 0.05,  ^∗∗^*p* < 0.01,  ^∗∗∗^*p* < 0.001, and^∗∗∗∗^*p* < 0.0001.

To confirm that tRF-5009A regulates autophagy and degeneration of the OA cartilage by targeting mTOR, we knocked down the latter using mTOR shRNAs. To determine which mTOR knockdown (KD) shRNA (KD-01/02/03/04) was the most effective in OA cartilage compared with knockdown negative control (KD-NC), we performed qRT-PCR analysis and found that mTOR KD-02 had the highest efficiency (Figure 7(a)). Thus, we used mTOR KD-02 in subsequent experiments. Our results revealed that cotransfection of cells with mTOR KD-02 and the tRF-5009A inhibitor significantly reversed the changes downstream of mTOR, increased the levels of COL2A1, ALs, and APs, but decreased those of MMP13, ROS, and cell apoptosis. Conclusively, we observed that mTOR KD-02 blocked the tRF-5009A inhibitor-mediated upregulation of mTOR (Figures 7(b)–7(j)).

### 3.7. Inhibition of Luciferase Reporter Activity of the 3′-UTR of *mTOR* mRNA by tRF-5009A

To demonstrate the mechanism underlying the regulation of the expression of mTOR by tRF-5009A, we analyzed the 3′-UTR sequence of human mTOR mRNA. We used the predictive bioinformatic programs miRanda and TargetScan to determine whether the 3′-UTR of mTOR contained a potential tRF-5009A-binding site. In addition, we performed a dual-luciferase reporter assay to determine whether the 3′-UTR of mTOR contained a tRF-5009A interaction sequence and subsequently mutated the ACAGAAG sequence into UCUGCAC (Figure 7(k)). We observed that the luciferase activity of wild-type mTOR varied substantially when tRF-5009A was overexpressed in cells. In contrast, the mutated binding sequence did not alter the mTOR 3′-UTR reporter activity when tRF-5009A was overexpressed (Figure 7(l)). These results confirmed that tRF-5009A reduced the luciferase activity by binding to the 3′-UTR of mTOR.

## 4. Discussion

An increasing number of studies have shown that tRNAs and tRFs play important roles in various cells [9, 10, 32]. tRFs are small noncoding RNAs (ncRNAs) that were discovered over the last few years. The implementation of novel improved techniques used for the sequencing of tRFs revealed that miRNA-like tRNA fragments are highly abundant in various cell types [33, 34]. For instance, tRF-3003a was identified by sequencing of the OA cartilage in a previous study [11]. In this study, we found that tRF-5009A was downregulated in OA ACL cells. OA is considered an intra-articular disease, characterized by cartilage degeneration, synovial membrane and infrapatellar fat pad inflammation, meniscus damage, and other important structural pathological changes [35]. Therefore, we explored the differences in the expression of tRF-5009A between OA and non-OA joint tissues.

Interestingly, we found that tRF-5009A was downregulated most significantly in the cartilage of patients with OA and was associated with certain clinical factors. Hence, we further investigated the expression of tRF-5009A in damaged and undamaged areas of the cartilage and found that it can be used to predict OA severity, especially in the Kellgren-Lawrence grade. Considering that OA is related to many pathogenic factors, including age, sex, obesity grade, and Kellgren-Lawrence grade, we explored the correlation between the level of expression of tRFs with these factors and further performed a regression analysis of influencing factors. As most patients with end-stage OA undergoing total knee arthroplasty are middle-aged or elderly, we enrolled 30 patients with knee OA aged 54 to 86 years. Therefore, in this study, we established 2 groups: the relatively young group (<65 years old) and the older group (≥65 years old). However, there was no significant difference in the expression of tRF-5009A between these 2 groups. This demonstrated that the expression of tRF-5009A is not necessarily associated with aging. Nonetheless, we found that the expression of tRF-5009A was positively correlated with obesity.

We revealed that the expression of the tRNA^ValCAC^ derived fragment tRF-5009A promoted autophagy and suppressed cartilage degeneration in OA via inhibition of the mTOR pathway. Autophagy plays an important role in a variety of diseases, including cartilage degeneration [36, 37]. Recent evidence has suggested that autophagy stabilizes the chondrocyte extracellular matrix, increases anabolism, but decreases catabolism, apoptosis, and ROS production [12, 38]. Several studies have shown that mTOR signaling is involved in the activation of autophagy in chondrocytes in OA [36, 39, 40]. Inhibition of the activity of mTOR improves autophagy by directly phosphorylating the serine sites of ULK1, an important regulatory protein for autophagy induction [41]. This inhibition effectively elevates autophagic flux, including the levels of Beclin1 and LC3 II. Previous studies have indicated that some microRNAs induce autophagy by inhibiting mTOR [42, 43]. Recent studies have also shown that tRF3008A suppresses the progression and metastasis of colorectal cancer by destabilizing FOXK1 in an AGO-dependent manner [9], while tRF-3001b aggravates the development of nonalcoholic fatty liver disease by inhibiting autophagy via the same mechanism [44]. Therefore, we hypothesized that tRF-5009A silences genes via base pairing with *mTOR* mRNA. In a previous study, tRF-3003a was identified by sequencing of the OA cartilage [11]. They found that a specific tRF, namely, tRF-3003a, can posttranscriptionally regulate JAK3 expression via AGO/RISC formation in chondrocytes. However, they did not explore the expression pattern of the tRF in human OA cartilage specimens and clinically relevant influencing factors as this work did. We firstly used more clinical samples to explore the differences of the tRF and explored the functions of the tRF in regulating chondrocyte autophagy in OA, which provided an additional tool for the clinical diagnosis and novel targeted therapies of OA.

## 5. Conclusions

**Figure 8:**
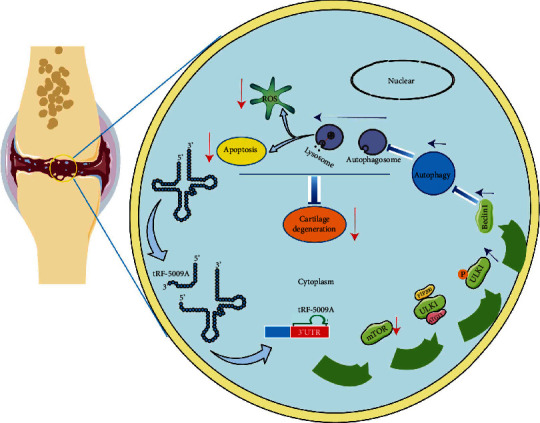
Schematic showing the proposed mechanism of suppression of cartilage degeneration through the regulation of autophagy by the binding of tRF-5009A to the 3′-UTR of mTOR in OA.

In conclusion, we demonstrated that tRF-5009A promoted autophagy and suppressed cartilage degeneration in OA by inhibiting mTOR (Figure 8). To the best of our knowledge, this is the first study to demonstrate a relationship between autophagy and tRFs in OA. These findings provide a new direction for the study of cartilage degeneration and the pathophysiological process of OA, thus providing an additional tool for the clinical diagnosis and novel targeted therapies of OA.

## Figures and Tables

**Table 1:** Baseline characteristics of patients with knee osteoarthritis (*n* = 30).

Characteristics	Number of cases (%)
Age (years)	
<65	11 (36.7)
≥65	19 (63.3)
Gender	
Male	6 (20.0)
Female	24 (80.0)
Obesity gradation^a^	
Underweight	0 (0.0)
Normal weight	9 (30.0)
Overweight	12 (40.0)
Obesity	9 (30.0)
Affected side	
Left	16 (53.3)
Right	14 (46.7)
Disease duration (years)	
<10	18 (60.0)
≥10	12 (40.0)
Kellgren-Lawrence gradation	
III	13 (43.3)
IV	17 (56.7)
Expression of tRF-5009A^b^	
Low expression	17 (43.3)
High expression	13 (56.7)
Expression of mTOR^c^	
Low expression	12 (40.0)
High expression	18 (60.0)

^a^Underweight: BMI < 18.5; normal weight: 18.5 ≤ BMI < 24; overweight: 24 ≤ BMI < 28; obesity: BMI ≥ 28. ^b^The maximal difference (eighteenth minus seventeenth is 0.0115 in ascending order) near median was used to classify between the low expression or high expression of tRF-5009A instead of the median (sixteenth minus fifteenth is 0.0002 in ascending order). ^c^The maximal difference (thirteenth minus twelfth is 0.0833 in ascending order) near median was used to classify between the low expression or high expression of mTOR instead of the median (sixteenth minus fifteenth is 0.0149 in ascending order).

**Table 2:** Correlation between tRF-5009A expression and the baseline characteristics of patients with knee osteoarthritis (*n* = 30).

Characteristics	tRF-5009A expression		*p* value
	Low	High	
Age (years)			
<65	5	6	0.454
≥65	12	7	
Gender			
Male	3	3	1.000
Female	14	10	
Obesity gradation			
Underweight	0	0	0.414
Normal weight	4	5	
Overweight	6	6	
Obesity	7	2	
Affected side			
Left	8	8	0.431
Right	9	5	
Disease duration (years)			
<10	10	8	0.880
≥10	7	5	
Kellgren-Lawrence gradation			
III	2	11	<0.001
IV	15	2	

*p* values were analyzed using the chi-squared test with *p* < 0.05 as significant.

**Table 3:** Spearman correlation analysis between tRF-5009A expression and the baseline characteristics of patients with osteoarthritis (*n* = 30).

Variables	tRF-5009A expression	
	Spearman	*p* value
Age	-0.271	0.148
Gender	-0.250	0.182
Weight	-0.377	0.040
Height	0.183	0.333
Body mass index	-0.490	0.006
Obesity gradation	-0.477	0.008
Affected side	-0.023	0.903
Disease duration	-0.173	0.361
Kellgren-Lawrence gradation	-0.727	<0.001

Note: *p* < 0.05 considered significant.

**Table 4:** Correlation between mTOR expression and the baseline characteristics of patients with knee osteoarthritis (*n* = 30).

Characteristics	mTOR expression		*p* value
	Low	High	
Age (years)			
<65	5	6	0.712
≥65	7	12	
Gender			
Male	3	3	0.660
Female	9	15	
Obesity gradation			
Underweight	0	0	0.422
Normal weight	5	4	
Overweight	5	7	
Obesity	2	7	
Affected side			
Left	7	9	0.654
Right	5	9	
Disease duration (years)			
<10	8	10	0.709
≥10	4	8	
Kellgren-Lawrence gradation			
III	10	3	0.001
IV	2	5	

*p* values were analyzed using the chi-squared test with *p* < 0.05 as significant.

**Table 5:** Spearman correlation analysis between mTOR expression and the baseline characteristics of patients with osteoarthritis (*n* = 30).

Variables	mTOR expression	
	Spearman	*p* value
Age	0.179	0.344
Gender	0.202	0.284
Weight	0.371	0.044
Height	-0.156	0.411
Body mass index	0.461	0.010
Obesity gradation	0.447	0.013
Affected side	0.023	0.903
Disease duration	0.185	0.328
Kellgren-Lawrence gradation	0.672	<0.001

Note: *p* < 0.05 considered significant.

## Data Availability

The research article data used to support the findings of this study are included within the article.

## Supplementary Materials

Supplementary Materials Supplementary Table S1: primer sequences for qRT-PCR.
