# Supplementary material for: tRNA-Derived Fragment tRF-5009A Regulates Autophagy and Degeneration of Cartilage in Osteoarthritis via Targeting mTOR
Source: Oxid Med Cell Longev. 2022 Aug 5;2022:5781660. doi: 10.1155/2022/5781660 (PMC9410839; doi:10.1155/2022/5781660)
Supplement: Supplementary Materials — Supplementary Table S1: primer sequences for qRT-PCR. [file 5781660.f1.docx]

Table S1: Primer sequences for qRT-PCR.

|  | Sequence |
| --- | --- |
| tRF-5009A | GGTGCTTCTGTAGTGTAGAAA |
| COL2A1-Forward primer | CCAGATGACCTTCCTACGCC |
| COL2A1-Reversed primer | TTCAGGGCAGTGTACGTGAAC |
| MMP13-Forward primer | CCAGACTTCACGATGGCATTG |
| MMP13-Reversed primer | GGCATCTCCTCCATAATTTGGC |
| mTOR-Forward primer | GCAGATTTGCCAACTATCTTCGG |
| mTOR-Reversed primer | CAGCGGTAAAAGTGTCCCCTG |
| U6-Forward primer | GGAACGATACAGAGAAGATTAGC |
| U6-Reversed primer | TGGAACGCTTCACGAATTTGCG |
| GAPDH-Forward primer | AGGTCGGTGTGAACGGATTTG |
| GAPDH-Reversed primer | GGGGTCGTTGATGGCAACA |
